# Death

**Published:** 1887-04-02

**Authors:** 


### Words of Consolation.

[Communications for this column should be addressed, "Chaplain," care of the Editor of The Hospital, who will gratefully welcome suggestions or inquiries from matrons, nurses, and the staff generally.]

### XXVII.—Death.

Why is death so often represented in Holy Scripture as sleep? The root idea of death in the Bible is generally "separation." The more permanent the separation, the more real therefore does death become. The first death consists in the separation of the soul from the body. The second death is the separation of the whole being of man from God. God does not count as dead those whose souls are only removed from their bodies for a short time. Such a separation is indeed a faint image, but no more, of the real death, which was present continually to our Lord's mind. He came to save us from the second death, not from the first. When, therefore, Christ or His Apostles spoke merely of physical death, they preferred calling it sleep. They could not apply a term which expressed the awful truth about hell to the translation of God's people from one sphere to another. The Scriptural, and therefore the Divine, idea of death points to it as the end of sin which finally involves the whole being and person of the sinner. "The soul that sinneth it shall die." "Why will ye die, O house of Israel?" "I have no pleasure in the death of the wicked, saith the Lord" (Ezek. xviii, 4, 19, 32). "I say unto you, my friends, Be not afraid of them that kill the body, and after that have no more that they can do. But I will forewarn you whom ye shall fear: Fear him, which after he hath killed, hath power to cast into hell: yea, I say unto you, fear him" (Luke xii, 4, 5). Take most of the passages in which God Himself speaks about death, and you will nearly always find they concern the death from which Christ came to deliver us, seldom the temporary penalty of soul and body being separated. As the Gospel revelation of life came to be unfolded, the followers of Christ spoke as He did. Just what we at this distance of time would characterise as a violent death (such was the end in this world of the martyr Stephen, and of many who followed in the same way), that the disciples called "falling asleep" (Acts vii, 60). Take any of the modern sensational accounts, the world's ideas of death, which flood the newspapers and literature of our time: these will be found as untrue to the Bible revelation as they are unchristian, and only too fully savoured with the offscouring of morbid and unfaithful minds. Compare these, such at least as you hope concern the dissolution of Christians, with the inspired accounts of St. Stephen, Lazarus, or Jairus's daughter, and what a contrast is presented! Is it not some consolation for those who have grown up, even in a Christian country, slowly imbibing a morbid untimely fear of death, who shrink from it, not so much on account of unforgiven sins, but rather because they have false notions of its supposed horrors, to fall back on their Saviour's assurance? "Fear not; only believe." "She is not dead but sleepeth." "Our friend Lazarus sleepeth." "Lord, lay not this sin to their charge: and when he had said this he fell asleep."

